# A cross-sectional study of community perceptions of stigmatization amongst women affected by UN-peacekeeper perpetrated sexual exploitation and abuse

**DOI:** 10.1186/s12889-021-12221-6

**Published:** 2021-12-18

**Authors:** Samantha Gray, Susan A. Bartels, Sabine Lee, Heather Stuart

**Affiliations:** 1 Department of Public Health Sciences, Queen’s University, Kingston, ON Canada; 2 Department of Emergency Medicine, Queen’s University, Kingston, Canada; 3 Department of History, University of Birmingham, Birmingham, England

**Keywords:** Democratic Republic of Congo, stigma, sexual exploitation, sexual abuse, peacekeepers, United Nations

## Abstract

**Background:**

Sexual exploitation and abuse (SEA) by UN peacekeepers perpetrated against local women and girls is a concern in the Democratic Republic of Congo (DRC). While stigma associated with sexual and gender-based violence is well documented more broadly, little is known about stigma associated with peacekeeper-perpetrated SEA.

**Methods:**

The aim of this study was to examine how the degree of exposure to SEA affects community perceptions of a woman or girl’s (1) social status (public stigma) and (2) institutional support in her community (structural stigma). Two poisson regression models with robust variance estimation were constructed utilizing community survey data of SEA experiences from eastern DRC (*n* = 2867) to quantify these associations. Relevant demographic variables were assessed for confounding and effect modification.

**Results:**

The prevalence of public and structural stigma were 62.9 and 19.3% respectively across the sample. A positive relationship was demonstrated between level of exposure of SEA and diminished social status in which women and girls experiencing moderate levels of SEA were at the greatest risk of public stigmatization after adjusting for confounding (RR: 1.94; CI: 1.66–2.26). Similarly, a positive relationship between exposure to SEA and inadequate institutional support was shown for female narrators wherein women and girls experiencing a high degree of SEA were 6.53 times as likely to receive inadequate support (RR: 6.53; CI: 3.63, 11.73). This contrasted with male narrated stories for whom there was no significant association between the SEA exposure level and institutional support.

**Conclusions:**

Women/girls with high exposure levels to UN peacekeeper-perpetrated SEA are at the highest risk of public and structural stigmatization, which should be more routinely considered when conceptualizing the consequences of SEA in peacekeeping contexts. The frequent occurrence of both public and structural stigma, coupled with the varying perceptions by sex, demonstrates the need for a multi-faceted approach for stigma reduction.

## Background

### Sexual Exploitation and Abuse by UN Peacekeeping Personnel

Sexual exploitation and abuse (SEA) have been widely reported in peacekeeping operations in Cambodia [1], Somalia [2], Haiti [3], Liberia, Kosovo, Sierra Leone, and Sudan [4]. Defined by the United Nations (UN) as “any actual or attempted abuse of vulnerability, differential power or trust, for sexual purposes, but not limited to, profiting monetarily, socially or politically from the sexual exploitation of another” ([5], p.6), sexual exploitation encompasses prostitution, transactional sex, and solicitation of transactional sexual interactions. Sexual abuse is defined as “an actual or threatened physical intrusion of a sexual nature, whether by force or under unequal or coercive conditions” ([5], p.5), with all sexual interactions involving minors under the age of 18 being considered abusive. Both within the UN and more broadly, SEA has been most often dichotomously measured with cases being examined against case criteria, and designated as SEA or not [6, 7]. This binary approach to measuring SEA fails to recognize that the physical, psychological and socioeconomic sequela may differ depending on the degree and nature of the event. Recent work has proposed a multi-dimensional measurement approach that would provide a more comprehensive and nuanced understanding of SEA that considers the dynamic and varied nature of women’s agency and victimization [8].

### Contributing Factors to UN-Perpetrated SEA in the DRC

Tasked with coordinating the peaceful transition of government and removal of armed forces from the Second Congo War, the United Nations Organization Mission in the Democratic Republic of Congo (MONUC) provided a moderate reduction in violence between 1999 to 2009 [9]. The mission was transformed into the United Nations Organization Stabilization Mission (MONUSCO) in 2010 to focus on the stabilization of government and protection of civilians, and continues today as the world’s largest UN peace support operation (PSO) [10]. Amongst the 32 publicly identified missions with SEA allegations, MONUC and MONUSCO are cited as contributing 26.9% of the total reports with 202 allegations [11]. It is important to note, however, that each allegation represents an uncorroborated report within which one or more perpetrators or victims are specified [11]. Since allegations may include multiple women, and sexual violence is historically underreported [12, 13], it is also important to note that the true number of host community members affected by peacekeeper-perpetrated SEA is likely higher.

Numerous factors are believed to increase the prevalence of UN-perpetrated SEA in particular settings. For instance, missions with larger numbers of uniformed peacekeepers and missions hosted in poorer countries with a lower GDP per capita, have been particularly associated with a higher frequency of SEA reports [4]. DRC is a powerful example of this since MONUSCO represents the largest ongoing mission in the world with approximately 18,500 troops, staff, police and volunteers [10]. Additionally, DRC retains a low-income status ($545.2 GDP per capita) [14, 15] and 77.1% of the country’s 81.3 million residents live below the income poverty line at a purchasing power parity of $1.90 USD a day [16]. The combination of local poverty and the authoritative presence of peacekeeping personnel is known to intensify power differentials between peacekeepers and the local population, in turn increasing the risk of coercion and transactional sex [12]. As peacekeepers have considerable access to resources such as money, weapons, and vehicles, their influence exceeds that of a local community member. Furthermore, unlike Congolese police and soldiers, peacekeepers are largely foreigners who are repatriated after their mission, presenting further challenges for survivors to access justice. Inequitable gender norms in the DRC and limited opportunities for justice for sexual violence survivors, in turn contribute to an environment of impunity in this population [16, 17].

### Stigmatizing Consequences of SEA

Physical injuries, sexually transmitted infections, unplanned pregnancies and psychological disorders are known consequences of sexual and gender-based violence. Negative social consequences of SEA, including community isolation [18–21], have been increasingly recognized in the literature, with stigma believed to play a key role [9, 19, 20, 22–26].

Stigma is the devaluation of a specified group because of directed negative beliefs and actions and can be categorized as self-stigma, public stigma, and structural stigma [27–29]. Self-stigma refers to an individual’s own devaluation of self and is often characterized by feelings of lack of confidence and low self-esteem [30]. Public stigma, in contrast, involves the devaluation of individuals by society in which the stigmatized individuals are isolated as ‘others’ and face unjust treatment or segregation by society [27]. Lastly, at a structural level stigma can be conveyed through institutional policies that restrict others by creating and maintaining social inequalities [27, 29].

Manifestations of public stigmatization are well reported among victims of sexual violence in the DRC [9, 19, 20, 22–26, 31]. Sexual violence survivors are often subjected to a devalued social status and/or are socially isolated from their communities and families leading to languishing mental health, fear of disclosing the incident, a loss of livelihood, forced relocation, and/or avoidance of medical services [17, 18, 20, 32, 33]. These consequences may also extend to interactions of transactional sex or sex work where women are labelled as ‘contaminated’ due to a fear of acquiring HIV/AIDS or other STIs [34, 35]. Additionally, some evidence suggests that structural stigmatization occurs for victims in the form of a lack of institutional protections, continuous impunity, breached confidentiality, and fear of reprisal from mental health and justice services [18, 20, 24].

While victims of sexual violence are recognized as being susceptible to stigmatization, a more nuanced understanding of stigma experiences among women and girls affected by peacekeeper-perpetrated SEA is needed. As stated, peacekeepers are uniquely characterized by their foreigner status and subsequent repatriation, considerable authority, and financial influence. These attributes of perpetrators remain unaccounted when examining stigmatization related to experiences of sexual violence in this population. Furthermore, the association between the level of exposure to SEA and experience of stigmatization has yet to be explored. This is despite accounts of varying experiences of SEA such as non-consensual sex and sex work being cited in literature [36], and the common usage of exposure-based measurement of event type and frequency in physical trauma literature to illustrate survivors’ experiences [37, 38]. It may be, for example, that a higher degree of exposure, denoted by an exposure to multiple sexually exploitative/abusive events with peacekeepers generate a greater risk for stigmatization. It is important to understand this relationship so that the unintended consequences of PSO are more fully appreciated and interventions to address harmful stigmatizing attitudes and behaviours can be appropriately designed for those who are most vulnerable. Therefore, this study aims to examine how the degree of exposure to peacekeeper-perpetrated SEA affects a woman’s perceived (1) social status and (2) institutional support in her community in the DRC.

## Methods

### Data Source

This study is a secondary analysis of cross-sectional data collected in a 9-week period between June and August 2018. The original survey examined the nature of interactions between UN peacekeepers and local women and girls in the DRC (*n* = 2867). Using convenience samples, interviews were conducted around six MONUSCO bases in eastern DRC: Kisangani, Bukavu, Goma, Benia, Bunia, and Kalemie. Beyond the initial sample, snowball sampling was utilized to recruit others within a 30 km radius of each of the chosen UN bases. Locally trained research assistants approached participants in naturalistic settings such as transportation hubs and markets for survey recruitment. Males and females over the age of 13 were asked to audio-record a short story about the interactions of local women/girls with UN peacekeepers. Subsequently, participants were asked to interpret their narrative by responding to a series of pre-defined questions in addition to providing their own demographic information (as opposed to the demographics of the woman/girl in the story). Survey questions were developed by a team of English-speaking experts in the areas of children born of war, humanitarian crises, sociology, political science, SenseMaker methodology, and sexual and gender-based violence. Congolese colleagues from SOFEPADI contributed to the survey development, adding contextual knowledge and local insights. Data was collected with Sensemaker®, a mixed-methods narrative capture tool designed to measure complex social patterns [39]. One type of Sensemaker interpretation question, *dyads*, asked participants to plot their perspectives about the events in the story on a continuous scale between two extreme options. To reduce social desirability bias, the two options within each question were matched in tone (both positive, both negative, or both neutral). Prior to data collection, the survey questions were pilot tested among a sample of 24 Congolese community members in order to improve the clarity and nuances in the translation.

**Fig. 1:**
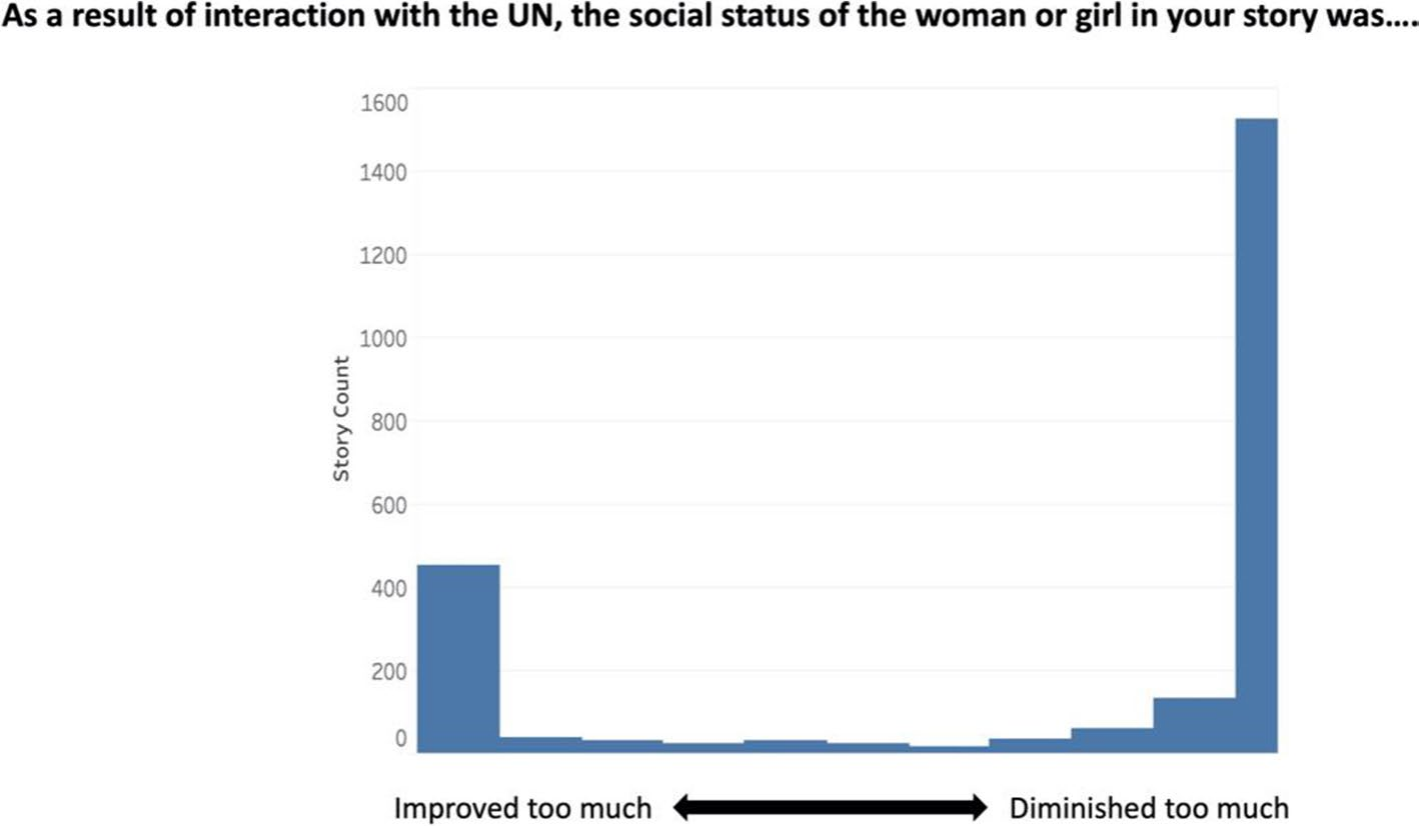
Dyad Representation of Social Status

Data points on the dyad hold a percentage value ranging from 0 to 100% for each of the two dimensions represented, and sum to 100%. Figure 1 demonstrates a dyad measuring the impact of the sexual interaction on the woman or girl’s social status. The extremes shown, both negative in tone, are ‘social status improved too much’ (dimension 1) or ‘social status diminished too much’ (dimension 2). A response at the leftmost extreme of the scale would express a value of 100% in the dimension of improved social status, and 0% in the dimension of diminished social status. Conversely, a response at the rightmost point of the dyad would indicate the opposite, a 100% value in the dimension of diminished social status, and 0% in the value of improved social status. If placed at the centre of the dyad, a 50% value would be assigned to each dimension, indicating no change in social status.

### Data Sampling

**Fig. 2:**
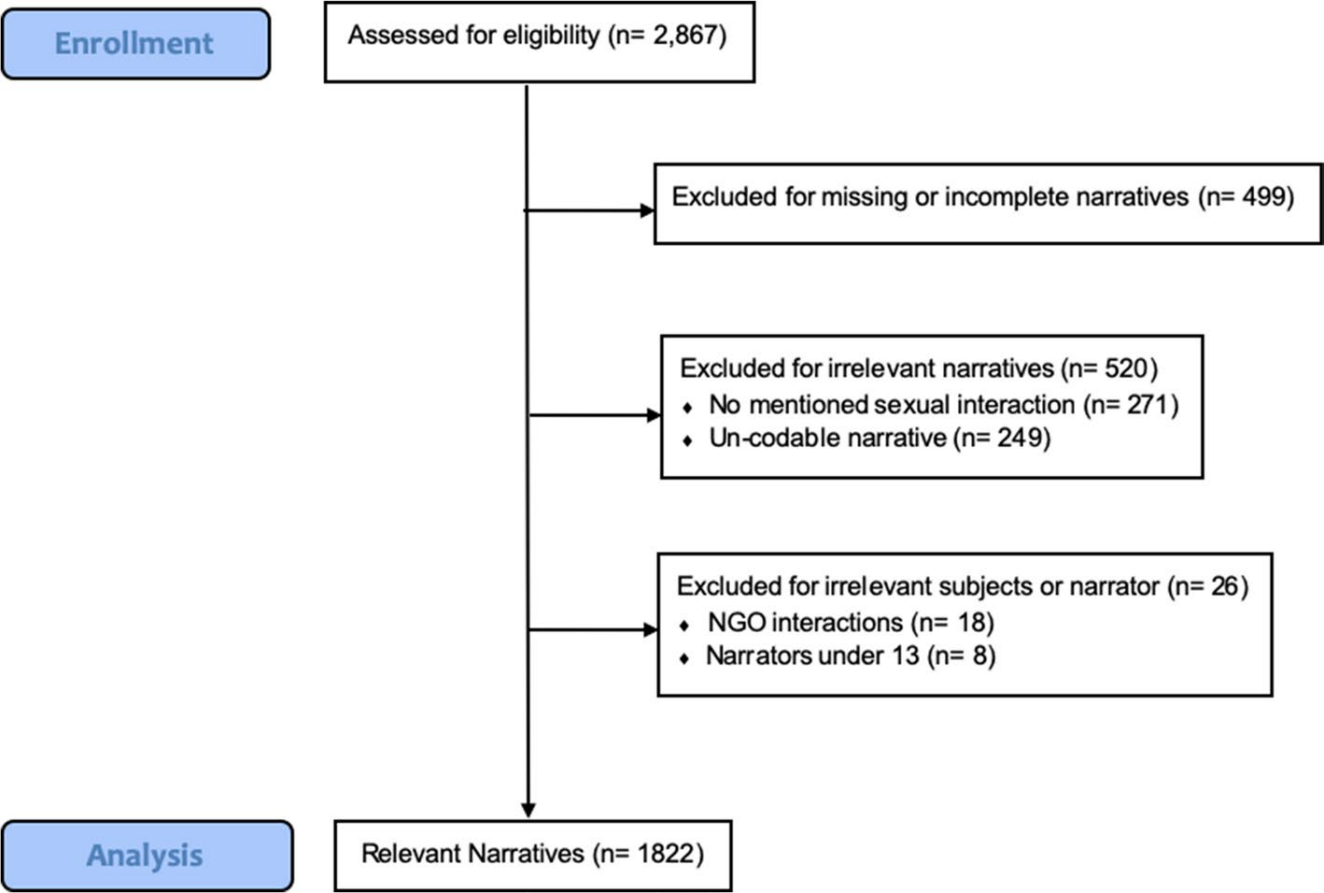
Data Sampling Flow Diagram

The data sampling strategy is outlined in Fig. 2. In total, 1045 narratives were excluded from the original dataset (*n* = 2867) on the basis of missing or incomplete stories (*n* = 499), irrelevant or un-codable narratives (*n* = 520) or excluded subjects/narrators (*n* = 26). Un-codable narratives were stories that described multiple sexual interactions involving several subjects. Such narratives were excluded as it was necessary that outcomes be attributable to only one subject. Data collected from participants under the age of 13 (*n* = 8) and sexual interactions involving NGO-workers rather than UN peacekeepers (*n* = 18) were removed as they were outside of the study’s objectives. In turn, 1822 narratives were utilized for analysis.

### Study Design

#### Exposure

The level of exposure to SEA was measured using a previously constructed 8-item index derived from the same study sample [8]. Participants were given a count for each UN peacekeeper-perpetrated sexually exploitative/abusive event in the narrative. Items were identified by a thematic analysis of the narratives and assessment of the pre-existing survey variables. Possible scores for this measure ranged from 0 to 8 with a higher count indicating a greater level of exposure to SEA. Scores were additionally classified into broader categories of mild (defined as 1 to 2 events experienced), moderate (3 to 4 events experienced), and high exposure to SEA (5+ events experienced) to reflect a gradation of exposure with an outcome of 0 indicating a non-exploitative/abusive sexual interaction. Sexually exploitative/abusive interactions were characterized by: the peacekeeper as the primary beneficiary, the peacekeeper as the primary initiator, transactional sex, a non-consensual sexual act, a non-consensual sexual exposure, the occurrence of sex work, sexual encounters with underage girls, or an unsupported peacekeeper-fathered pregnancy.

#### Outcome Cut-Point Analysis

The two model outcomes were dichotomized according to their respective dyad distributions. Cut-points were placed at natural distributional breaks as indicated by Moore, Lippman, & Brown [40]. This method of dichotomization was utilized due to an absence of precedent indicators and relevant literature detailing the measurement of both outcomes.

#### Outcome 1: Diminished Social Status

**Fig. 3:**
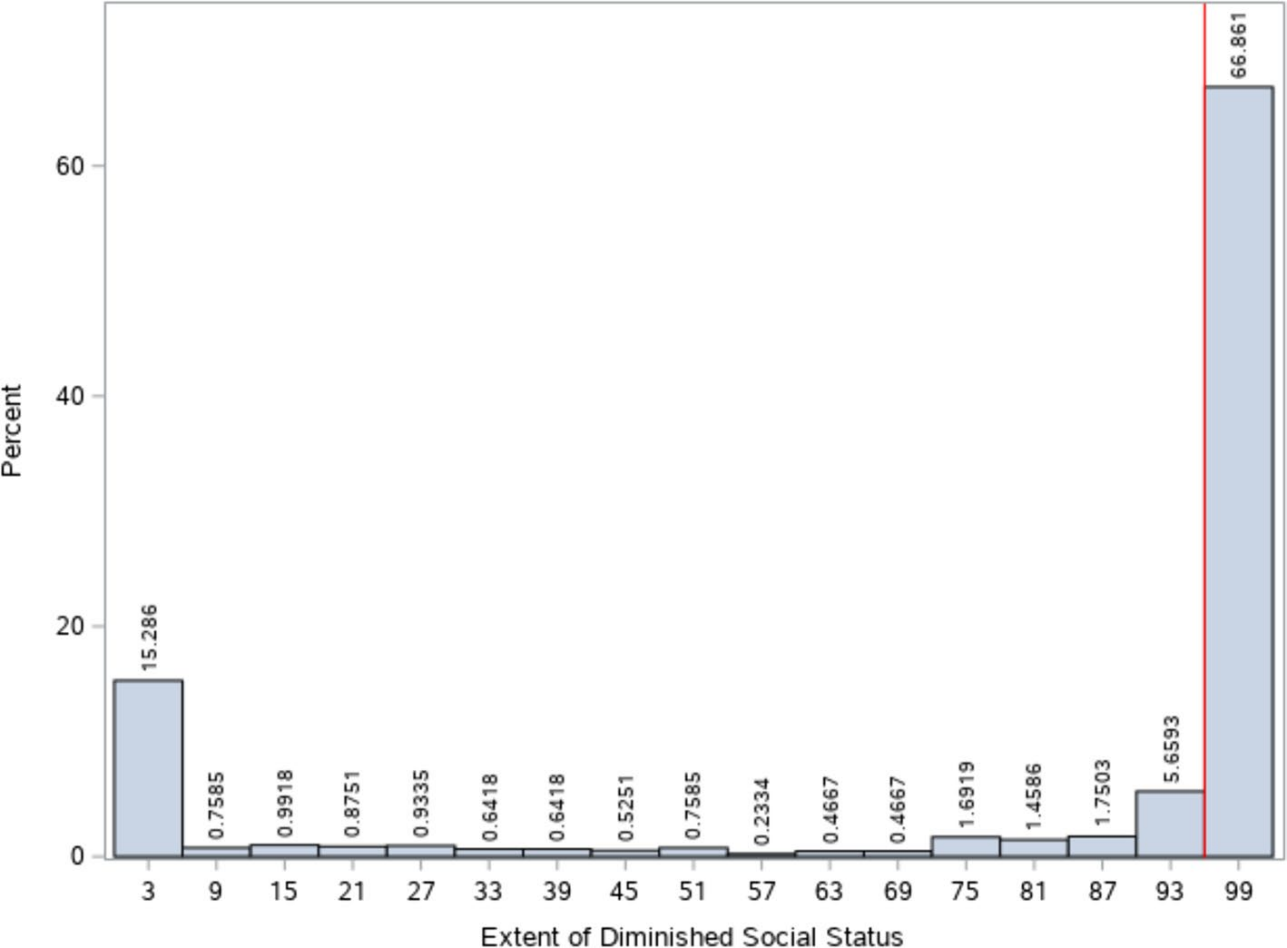
Frequency Distribution and Cut-Point for the Diminished Social Status Exposure

Diminished social status was defined as a perceived negative shift in the woman or girl’s public perception in her community. A dyad measuring the extent to which the woman or girl’s social status was improved too much (dimension 1) or diminished too much (dimension 2) as a result of her interaction with the UN peacekeeper was utilized for this measure. Participant responses on this dyad corresponded to numeric values from 0 to 100. Figure 3 outlines the distribution of responses and their corresponding numeric values in the dimension of diminished social status. A value of 0 indicated that ‘social status improved too much’ and a value of 100 indicated that ‘social status diminished too much’. A value of 50 indicated that no change in social status occurred as a result of the sexual interaction. Upon examining this frequency distribution, responses at or above the cut-point of 96 were considered to have a diminished social status. This point represented both a clear distributional break and indicator of a substantive reduction in social status.

#### Outcome 2: Inadequate Institutional Support

**Fig. 4:**
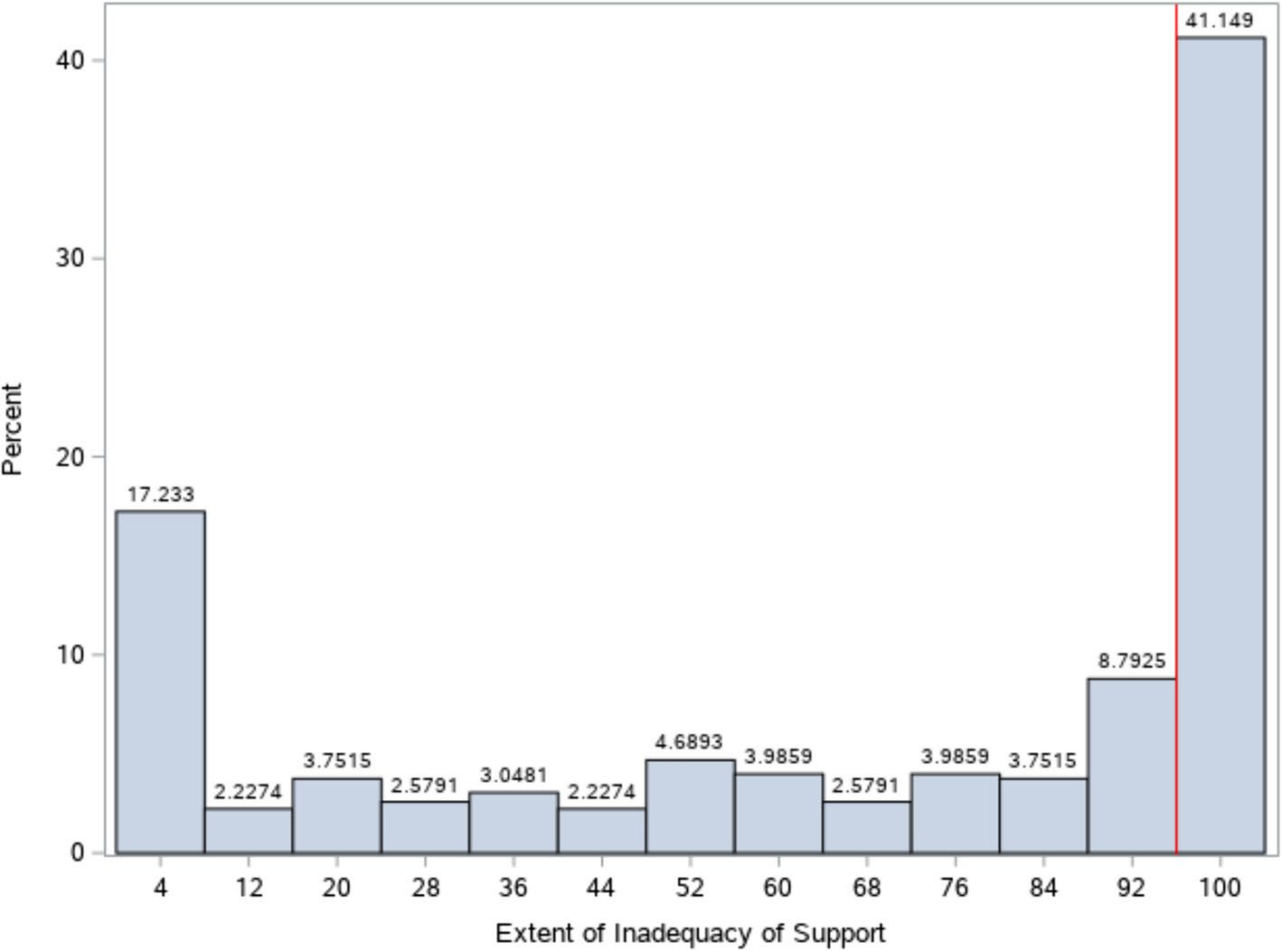
Distribution and Cut-Point for the Inadequate Institutional Support Indicator

Inadequate institutional support was defined as a perceived absence of or lack of support to the affected woman or girl by individuals in power. This was similarly measured using dyad responses. Participants were asked about the extent to which those in power did absolutely nothing to support the woman/girl (dimension 1) or provided the woman/girl with too much support (dimension 2). The interpretation of ‘support’ was left open to participants, and may have been considered as financial, health, job, justice, or housing based. Figure 3 outlines the distribution of responses and their corresponding numeric values in the dimension of inadequate support. A value of 0 indicated that ‘those in power provided her with too much support” and a value of 100 indicated that ‘those in power did absolutely nothing to support her’. A value of 50 indicated that enough support was given as a result of the sexual interaction. Similarly, responses at or above the chosen cut-point of 96 were indicated as receiving inadequate support (Fig. 4). This point represented a clear distributional break and indicator of a significant absence of institutional support.

#### Narrative Type

The type of narrative was measured by asking participants to identify the subject of the story. The options were listed as (a) about someone else I know, (b) about me, (c) about someone in the family, (d) about something I heard or read, or (e) prefer not to say. These categories were then collapsed into (a) first-person narrative or (b) third-person narrative for the purposes of this analysis.

#### Confounders and Effect Modifiers

Suspected confounders were grouped as characteristics of the narrator or characteristics of the sexual interaction. Narrator confounders included sex, age, socio-economic status, marital status, highest educational level achieved, and geographic location at the time of study participation. Sexual interaction confounders included the peacekeeper’s personnel role, nationality, and whether the story mentioned a peacekeeper fathered child or ‘peace-baby’. Sex was additionally assessed for effect modification. Suspected confounders and effect modifiers are outlined in Table 1.

**Table 1:** Demographic Characteristics of the Sample

Characteristic		Frequency
Sex		***N*** **= 1819**
Female		947 (52.1%)
Male		872 (47.9%)
Age		***N*** **= 1812**
Underage	13–17	139 (7.7%)
Adult	18–24	667 (36.8%)
	25–34	632 (34.84%)
	35–44	237 (13.1%)
	45–54	90 (5%)
	55–64	40 (2.2%)
	65 and Older	9 (0.5%)
Socio-Economic Status		***N*** **= 1820**
Low (0–1 items)		506 (27.8%)
Moderate (2–3 items)		822 (45.2%)
High (4–5 items)		492 (27%)
Highest Education Achieved		***N*** **= 1814**
No Formal Schooling		112 (6.2%)
Primary School		330 (18.17%)
Secondary School		751 (41.4%)
Technical Training / University		615 (39.4%)
Other		6 (0.3%)
Marital Status		***N*** **= 1816**
Single Never Married		976 (53.6%)
Married /Living with Partner		690 (37.9%)
Separated / Divorced		90 (5%)
Widowed		60 (3.3%)
Geographic Location		***N*** **= 1820**
Population of 500, 000 or more (65.66%)	Bukavu	248 (13.6%)
	Goma	337 (18.5%)
	Kalemie	163 (8.95%)
	Kisangani	449 (24.6%)
Population under 500, 000 (34.34%)	Beni	208 (11.4%)
	Bunia	417 (22.9%)

#### Narrator’s Sex

Sex was measured as (a) female, (b) male or (c) prefer not to say.

#### Narrator’s Age

Age was measured as (a) under 13, (b) 13 to 17, (c) 18 to 24, (d) 25 to 34, (e) 35 to 44, (f) 45 to 54, (g) 55 to 64, (h) 65 and older, or (i) prefer not to say. However, these categories were collapsed into (a) 13 to 17, (b) 18 to 24, and (c) 25 and older for the purposes of analysis.

#### Narrator’s Socio-Economic Status

Socio-economic status (SES) was measured as a score from 0 to 5 according to the number of items possessed in the household: a mobile phone, radio, generator inverter or sun panel, a vehicle (i.e. motorbike or car), and a refrigerator or freezer. This was condensed further into low (0–1 items), moderate (2–3 items), and high (4–5 items) socio-economic groups.

#### Narrator’s Marital Status

Marital status at the time of the survey was measured as (a) single never married, (b) married/living with partner, (c) separated/divorced, (d) widowed, or (e) prefer not to say.

#### Narrator’s Highest Education Achieved

The highest level of education achieved by the narrator was measured by an 11-part multiple choice question detailing levels of primary, secondary, technical, and university-level training. However, these categories were modified for analysis as (a) no formal schooling, (b) some or all of primary school, (c) some or all of secondary school, (d) some or all of technical training or university, or (e) other.

#### Narrator’s Urban Status

The urban status of the narrator was derived from a variable measuring the geographic location of the sample collection by city (Bukavu, Goma, Kalemie, Kisangani, Beni, and Bunia). These cities were dichotomized by population size (a) greater or equal to 500,000 or (b) less than 500,000, for the assessment of confounding to determine if community size was a contributing factor. These categories were created in accordance to the intermediary and small cities classifications by population size utilized by the United Cities and Global Governments [41].

#### Peacekeeper’s Role

The peacekeeper’s role within the PSO was derived from participants’ responses when asked that question directly with possible options including (a) an armed soldier, (b) a UN civilian, (c) an unarmed soldier, (d) UN police, (e) don’t know or (f) other. For the purposes of analysis, these categories were collapsed as (a) soldier (armed or unarmed), (b) non-soldier (civilian or police), or (c) unspecified.

#### Peacekeeper’s Nationality

Nationality was identified by asking participants to recall the flag on the peacekeeper’s uniform and match it to a labeled flag on the questionnaire. The following were listed as possible responses: (a) Bangladesh, (b) DRC, (c) Egypt, (d) Ghana, (e) India, (f) Morocco, (g) Nepal, (h) Pakistan, (i) South Africa, (j) Senegal, (k) Sierra Leone, (l) Tanzania, (m) Uruguay, (n) don’t know or (o) other. For this analysis, countries were grouped more broadly by continent as (a) African, (b) Asian, (c) South American, or (d) unspecified.

#### Narrative About Peacekeeper-Fathered Child

Research assistants identified narratives as being (a) about a peacekeeper-fathered child, (b) mentioned a peacekeeper-fathered child, or (c) neither. Stories about or mentioning a peacekeeper-fathered child were combined for this measurement.

### Study Analysis

Analyses were performed using SAS software version 9.04. Univariate descriptive analyses were performed to provide a demographic profile of the study sample, and characteristics of the narratives. Chi-square tests were utilized for the bivariate analysis of covariates against the SEA index exposure, diminished social status and inadequate support outcomes. Two Poisson regression with robust variance estimation models were constructed using an effect estimation approach with consideration to possible confounding and effect modification. Manual backwards elimination was utilized for variable removal with a liberal *p*-value criterion of 0.15. Confounders and effect modifiers were tested a-priori by chi-square analyses to determine if each was associated with the SEA exposure and stigma model outcomes. A sensitivity analysis was conducted to gauge the reliability of the third-person narratives when assessing the relationship between exposure to SEA and stigmatization. Relative risk estimates were compared between adjusted models with and without the inclusion of narrative type.

## Results

The demographic characteristics of the sample are outlined in Table 1. In total, 52.1% of the stories were shared by female narrators, and 44.5% of the sample was under the age of 25. Approximately half of the sample was classified at a moderate socio-economic level (45.2%), 65.7% lived in a city with a population greater than 500,000 and 80.8% reported attaining a secondary or higher education. In terms of marital status, 53.6% of the sample reported being single and never married. Table 2 outlines the characteristics of the micro-narratives. Third-person narratives, reflecting community perceptions of stigmatization, composed the vast majority of the sample at 87.6%. Peacekeeper-fathered children were mentioned in 53.2% of stories, and ‘other’ was the most frequently cited peacekeeper nationality (19.3%).

**Table 2:** Characteristics of the Narrative

Story Perspective		***N*** **= 1818**
First-Person		226 (12.4%)
Third-Person		1592 (87.6%)
Peacekeeper’s Role		***N*** **= 1819**
Soldier	Armed	1035 (56.9%)
	Unarmed	266 (14.6%)
Non-Soldier	UN Civilian	288 (15.8%)
	UN Police	38 (2.1%)
Unspecified	Don’t Know	142 (7.8%)
	Other	50 (2.8%)
Peacekeeper’s Nationality		***N*** **= 1822**
African	DRC	103 (5.7%)
	Egypt	69 (3.8%)
	Ghana	30 (1.6%)
	Morocco	187 (10.3%)
	South Africa	343 (18.8%)
	Senegal	138 (7.6%)
	Sierra Leone	10 (0.6%)
	Tanzania	197 (10.8%)
Asian	Bangladesh	101 (5.5%)
	India	125 (6.9%)
	Nepal	71 (3.9%)
	Pakistan	83 (4.6%)
South American	Uruguay	200 (11%)
Unspecified	Other	352 (19.3%)
	Don’t Know	236 (12.9%)
Story About a Peacekeeper Fathered Child		
About or Mentioned a Peacebaby		970 (53.2%)
Neither		852 (46.8%)

The mean SEA exposure score was 1.6 ± 1.1 with two participants reporting the highest demonstrated score of 6 (Table 3). Approximately 15% of participants reported no sexually abusive or exploitative events. A noticeable difference in the prevalence of the stigma outcomes was noted with 62.9% of participants reporting a diminished social status, and 19.3% reporting inadequate institutional support for the woman or girl in the story.

**Table 3:** Frequency of Exposure and Outcome Amongst Sample

SEA Exposure Score	***N*** **= 1824**
0	270 (14.8%)
1	680 (37.3%)
2	536 (29.4%)
3	243 (13.3%)
4	71 (3.9%)
5	20 (1.1%)
6	2 (0.1%)
Mean	1.6 ± 1.1
Median	1
Interquartile Range	1
Diminished Social Status	***N*** **= 1824**
Yes	1146 (62.9%)
No	678 (37.1%)
Inadequate Community Support	***N*** **= 1824**
Yes	351 (19.3%)
No	1473 (80.7%)

Bivariate analyses between the covariates and the degree of exposure to SEA, as well as between the covariates and the two stigmatization outcomes revealed significant correlations (Table 4). The sex of the narrator, area collected, and peacekeeper’s role were identified as potential confounders for both models. The martial status of the narrator was highlighted as a potential confounder for the diminished social status model while only the socio-economic status and narrator educational attainment were potential confounders for the inadequate support model.

**Table 4:** Bivariate Relationships of Variables

Variable	Exposure to SEA (Exposure)	Social Status (Outcome)	Anticipated Effect for Model 1	Community Support (Outcome)	Anticipated Effect for Model 2
Sex	χ^2^ = 42.5713 df = 6 *p* = <0.0001*	χ^2^ = 11.9938, df = 1 *p* = 0.0005*	Confounder	χ^2^ = 4.9654 df = 1 *p* = 0.0259*	Confounder
Age	χ^2^ = 19.2536 df = 12 *p* = 0.1826	χ^2^ = 1.6275 df = 2 *p* = 0.4432	Insignificant effect	χ^2^ = 14.8743 df = 2 *p* = 0.0006*	Predictor of outcome
SES	χ^2^ = 31.0278 df = 12 *p* = 0.002*	χ^2^ = 2.1734 df = 2 *p* = 0.3373	Predictor of exposure	χ^2^ = 42.5850 df = 2 *p* = <0.0001*	Confounder
Education	χ^2^ = 36.4445 df = 24 *p* = 0.0497*	χ^2^ = 6.9128 df = 4 *p* = 0.1406	Predictor of exposure	χ^2^ = 61.3297 df = 4 *p* = <0.0001*	Confounder
Marital Status	χ^2^ = 44.2014 df = 24 *p* = <0.0072*	χ^2^ = 13.3589 df = 4 *p* = 0.0096*	Confounder	χ^2^ = 7.0117 df = 4 *p* = 0.1353	Predictor of exposure
Area Collected	χ^2^ = 53.40 df = 6 *p* = <0.0001*	χ^2^ = 13.8887 df = 1 *p* = 0.0002*	Confounder	χ^2^ = 106.6403 df = 1 *p* = <0.0001*	Confounder
Peacekeeper’s Role	χ^2^ = 57.5814df = 24 *p* = 0.0001*	χ^2^ = 32.9425 df = 4 *p* = <0.0001*	Confounder	χ^2^ = 26.4622 df = 4 *p* = <0.0001*	Confounder
Peacekeeper’s Nationality	χ^2^ = 60.5565 df = 24 *p* = 0.0001*	χ^2^ = 3.3366 df = 4 *p* = <0.5032	Predictor of exposure	χ^2^ = 7.5169 df = 4 *P* = 0.1110	Predictor of exposure
Story About a Peacekeeper Fathered Child	χ^2^ = 19.43 df = 6 *p* = 0.0035*	χ^2^ = 4.53 df = 1 *p* = 0.0334*	Confounder	χ^2^ = 4.16 df = 1 *P* = 0.0414*	Confounder

* Statistically significant correlation

Table 5 outlines the crude and adjusted Poisson regression estimates for model 1 examining change in social status. Both models demonstrated a positive relationship between the SEA exposure level and social status with the likelihood of perceived public stigmatization increasing with higher SEA scores. This risk peaked at a moderate SEA exposure score of 4 with 2.11 times the risk of public stigmatization in the crude model (RR: 2.11; CI: 1.81–2.46) and plateaued with SEA scores of 5 or more (RR: 1.99; CI: 1.61–2.47). When adjusted for the narrator’s marital status, education level, population size, and the UN personnel’s role, woman/girls with SEA scores of 4 were 1.94 times as likely to be publicly stigmatized in comparison to non-exploited/abused counterparts (RR: 1.94; CI: 1.66–2.26).

**Table 5:** Poisson Regression Estimates of Diminished Social Status (Model 1)

SEA Exposure Score	Unadjusted Model^a^ RR (95% CI)	Adjusted Model^b^ RR (95% CI)
0	1.00 (ref)	1.00 (ref)
1	1.18 (1.01, 1.38)	1.14 (0.98, 1.33)
2	1.69 (1.46, 1.96)	1.59 (1.38, 1.84)
3	1.95 (1.68, 2.26)	1.82 (1.57, 2.11)
4	2.11 (1.81, 2.46)	1.94 (1.66, 2.26)
5+	1.99 (1.61, 2.47)	1.88 (1.52, 2.34)

^a^ Quasi-likelihood under Independence Model Criterion (QIC) = 8834.65

^b^Adjusted for the narrator’s marital status and highest level of education achieved, the population size of the area collected, and the UN personnel’s role. QIC = 8786.75

Crude and adjusted estimates for model 2, which examined access to support, are highlighted in Table 6. Due to evidence of effect modification, model estimates were stratified by the sex of the narrator. In female-narrated stories, crude and adjusted estimates of mild, moderate, and high exposure scores of SEA indicated an increased perceived risk of inadequate institutional support. This risk was most substantial in women or girls with SEA scores of 5, who were 6.98 times as likely to receive inadequate support (RR: 6.98; CI: 3.66–13.32). After adjusting for demographic factors, scores of 5 or more were associated with a relative risk of 6.53 (CI: 3.63, 11.73).

**Table 6:** Poisson Regression Estimates of Inadequate Support (Model 2)

SEA Exposure Score	Unadjusted Model^a^ RR (95% CI)	Adjusted Model^b^* RR (95% CI)
	**Females (** ***n*** **= 947)**	
0	1.00 (ref)	1.00 (ref)
1	1.59 (0.95, 2.65)	1.72 (1.04, 2.86)
2	2.21 (1.33, 3.70)	2.65 (1.60, 4.40
3	1.88 (1.02, 3.45)	2.33 (1.29, 4.22)
4	4.12 (2.22, 7.66)	4.27 (2.21, 8.27)
5+	6.98 (3.66, 13.32)	6.53 (3.63, 11.73)
	**Males (** ***n*** **= 872)**	
0	1.00 (ref)	1.00 (ref)
1	0.70 (0.42, 1.15)	0.91 (0.57, 1.47)
2	1.43 (0.91, 2.25)	1.83 (1.19, 2.81)
3	1.01 (0.60, 1.71)	1.51 (0.92, 2.50)
4	1.56 (0.84, 2.91)	2.09 (1.19, 3.66)
5+	0.76 (0.19, 2.91)	1.13 (0.27, 4.72)

^a^ QIC_FEMALE_ = 1066.29; QIC_MALE_ = 1190.45

^b^Adjusted for the narrator’s socio-economic status, marital status, and highest level of education achieved as well as the population size of the area collected, and the UN personnel’s role. QIC_FEMALE_ = 1057.82; QIC_MALE_ = 1078.09

This pattern was not paralleled in male-narrated stories, as the crude model indicated no significant change in risk of inadequate support amongst all exposure levels. In the adjusted model, a perceived increased risk of structural stigmatization was observed exclusively for women and girls with SEA exposure scores of 2 (RR: 1.83; CI: 1.19–2.81) and 4 (RR: 2.09; CI: 1.19–3.66). A seemingly protective effect was present with male narrators only for SEA scores of 1 in both the crude and adjusted, and 5 in the crude model, however these estimates were not significant.

Upon comparison of relative risk estimates between the adjusted models of diminished social status and inadequate institutional support with and without accounting for narrative type, no significant differences were found. Tables 7 and 8 outline these comparisons respectively.

**Table 7:** Comparison of Adjusted Estimates of Diminished Social Status (Model 1)

Exposure Level	Original Adjusted Model	Model Adjusted for Narrative Type
0	1.00 (ref)	1.00 (ref)
1	1.14 (0.98, 1.33)	1.14 (0.98, 1.33)
2	1.59 (1.38, 1.84)	1.60 (1.38, 1.85)
3	1.82 (1.57, 2.11)	1.82 (1.57, 2.12)
4	1.94 (1.66, 2.26)	1.98 (1.69, 2.32)
5+	1.88 (1.52, 2.34)	1.97 (1.58, 2.45)

**Table 8:** Comparison of Adjusted Estimates of Inadequate Support (Model 2)

Exposure Level	Original Adjusted Model	Model Adjusted for Narrative Type
Females (*n* = 947)		
0	1.00 (ref)	1.00 (ref)
1	1.72 (1.04, 2.86)	1.70 (1.02, 2.83)
2	2.65 (1.60, 4.40)	2.60 (1.56, 4.32)
3	2.33 (1.29, 4.22)	2.27 (1.25, 4.12)
4	4.27 (2.21, 8.27)	4.13 (2.13, 8.00)
5+	6.53 (3.63, 11.73)	6.07 (3.29, 11.20)
Males (*n* = 872)		
0	1.00 (ref)	1.00 (ref)
1	0.91 (0.57, 1.47)	0.91 (0.56, 1.46)
2	1.83 (1.19, 2.81)	1.81 (1.18, 2.77)
3	1.51 (0.92, 2.50)	1.51 (0.91, 2.49)
4	2.09 (1.19, 3.66)	2.06 (1.18, 3.62)
5+	1.13 (0.27, 4.72)	1.11 (0.27, 4.68)

## Discussion

Model estimates suggest that female victims of peacekeeper-perpetrated SEA in the DRC experience both public and structural stigmatization. With frequencies of 62.9% (public) and 19.3% (structural), stigma was a relatively common outcome in this sample. General community perceptions and female-narrated stories indicated that women and girls who experienced a moderate to high degree of exposure to SEA were at the greatest risk of diminished social status and inadequate institutional support respectively.

Relative risk estimates for inadequate support among female participants were of the highest magnitude. However, this was contrasted by male-narrated stories of inadequate support, in which there was no perceived significant risk of stigmatization at any level of SEA exposure. In turn, it is suggested that female narrators held a unique recognition of the structural obstacles faced by women and girls who experienced SEA. Overall, these findings imply that public and structural stigma are significant consequences with sex being a significant factor in the perception of the latter. Additional research is needed to understand the mechanism behind this sex-based difference in perceptions.

The positive relationship between the level of exposure to SEA and stigmatization may be derived from societal perceptions and gender norms that hold women and girls responsible for these sexual encounters. This ideology is rooted in attribution theory which explains that individuals often counterintuitively assign blame to victims of crime because they are perceived as having behaved in a manner that contributed to the outcome [42, 43]. This is known to occur, for instance, with sexual violence victims who are often shamed for sexual assaults [42] and intimate partner violence [44]. Compared to victims of other traumatic events such as natural disasters or other interpersonal violence, sexual violence victims are alleged to be more at fault, less likeable and less credible when describing the events endured [45]. This perpetuation of blame is likely to negatively impact the woman or girl’s social status, and/or when she is deemed less credible, to affect her experience in receiving support services.

Additionally, an ingrained gendered component of attribution may explain the large gap in the female and male perceptions of institutional support inadequacy. Previous literature has indicated that perceptions of sexual violence and its effects are often derived from societal values around gender roles and sexuality [46] wherein more traditional gender beliefs are associated with a higher tolerance of perpetration towards women [47–49]. In the DRC, gender attitudes and inequality can be characterized as traditional in nature, with clear family roles in an environment where women often do not benefit from the same social, political, and economic status as men [50, 51]. Men are often designated as community leaders and principal decision makers in their families even with regards to matters related to women’s health [51]. As a result of these norms, men may act as social deterrents to accessing care and support services while underscoring other aspects of structural stigmatization faced by those affected by SEA [51, 52]. From this perspective, we believe that gender norms need to be considered carefully when conceptualizing peacekeeper-perpetrated SEA and how to address its associated stigma in the DRC.

The impact of stigma on a woman or girl’s livelihood can be comparable to the trauma of the sexual act(s) itself [53, 54]. As diminished social status and inadequate access to essential services are recognized as counterproductive for recovery [19], targeted interventions in these areas may improve the wellbeing of affected women and girls. Engagement with community leaders to influence public attitudes towards women’s health and access to services has been a successful strategy in African contexts [55, 56], and may be paralleled to influence perceptions of those affected by UN-perpetrated SEA. The current work highlights a need to engage with influential leaders, who are often male, to encourage male community members to recognize the need for support services to reduce this social constraint on services.

Furthermore, the high rates of public and structural stigmatization likely discourage affected women and girls from disclosing their experiences of peacekeeper-perpetrated SEA, which may negatively impact formal reporting to the UN. While UN public reporting mechanisms have improved through increased public awareness and geographic access [57], it is suspected that many SEA cases are still not captured in official estimates. Women and girls affected by UN-perpetrated SEA may be fearful of being blamed or discredited in their communities, as suggested by the high rates of stigma identified in this study, and that this may prevent them from engaging with the UN’s formal complaint system. The extent of inadequate institutional support, particularly with high exposure to SEA, suggests that structural barriers to accessing care and services are substantial and require broad policy-based change. Our research is intended to provide precursory knowledge for a multi-faceted approach to tackling stigmatization of women and girls at both the community and institutional level, and we recommend additional research to understand how stigma may contribute to under-reporting of SEA.

The increased risk of public and structural stigmatization revealed in this analysis is similar in pattern to the increased risk of community rejection and experiences of inadequate medical care denoted in female high school student survivors of sexual violence in the DRC [58]. Females aged 11 to 23 who had a non-consensual sexual experience or rape were 3.93 and 16.87 times as likely to experience rejection by their community in comparison to females with no experience of sexual violence [58]. Furthermore, females who had a non-consensual sexual experience or experienced rape were 1.66 and 4.41 times as likely to receive inadequate care respectively [58]. These patterns are similar in direction to the relative risk ranges of the diminished social status (RR: 1.14–1.88) and female-narrated inadequate support models with scores of 1 or more (RR: 1.72–6.53). Further research is needed to understand the experiences of public and structural stigmatization derived from sexual violence between peacekeeper and non-peacekeeper perpetrators in the DRC.

### Limitations

A few limitations should be noted. Firstly, the sample cannot be considered representative of MONUSCO-affected communities in the DRC as a result of the convenience sampling approach. The larger representation of third-person accounts is speculated to be attributed to a reluctancy for affected women/girls to participate due to fear of judgement for sharing their experiences with the research team. While sufficient reliability of the third-person narratives is assumed from the sensitivity analysis, this large portion of third-person narratives emphasizes the potential for recall bias to be a factor in this sample as participants were asked to recount previous experiences. It is suspected that first-person accounts could provide a more accurate recollection than accounts of another individual’s experience. In turn, recall bias may disproportionally affect third-person narratives causing a misclassification of the degree of exposure to SEA, particularly if the narrator relied upon gossip as the principal source of information for the narrative. As the index exposure is categorical, the effect on the relative risk estimates was likely biased to the null. Further validation of the SEA index measure through expert consultation is needed. Furthermore, while survey pilot testing was conducted among a Congolese community sample, cognitive testing for the stigmatization outcomes remains an area for future research. The dichotomization of the two outcome variables additionally reduces statistical power. Additionally, this analysis did not consider the impact of potential confounders such as previous exposure to SEA or generalized violence [19], as these factors were not measured in the original data sample.

### Strengths

To our knowledge, this study represents the first to examine the association between public and structural stigmatization and the degree of exposure to peacekeeper-perpetrated SEA. The recognition of public and structural stigmatization for women and girls as a consequence of experiencing SEA in this context at varying exposure levels is vital as it suggests the usage of a multi-faceted approach for intervention at both a community and institutional level. Additionally, this study recognizes that sex and associated gender norms may alter the perceptions of those affected peacekeeper-perpetrated SEA, and therefore should be considered in this context.

## Conclusions

This study highlights that women and girls who experience UN-peacekeeper perpetrated SEA are susceptible to public and structural stigmatization. In fact, diminished social status and inadequate institutional support are relatively common for affected women and girls in the DRC. In particular, women and girls highly exposed to SEA are at the highest risk of experiencing a diminished social status and/or inadequate institutional support, however a difference in perception of institutional support by sex demonstrates there is a gendered component at least in the DRC context that would benefit from targeted interference. The occurrence of both public and structural stigmatization and varying perceptions by sex argues for a multi-leveled approach for stigma reduction. In summary, it is recommended that interventions utilize male community leader engagement and policy-based measures to influence public attitudes and service accessibility affecting women and girls highly exposed to SEA.

## Data Availability

The dataset analysed during the current study is not publicly available at this time.

## Abbreviations

DRC: Democratic Republic of Congo
MONUC: United Nations Organization Mission in the Democratic Republic of the Congo
MONUSCO: United Nations Organization Stabilization Mission in the Democratic Republic of the Congo
NGO: Non-governmental organization
PSO: Peace support operation
SEA: Sexual exploitation and abuse
UN: United Nations
