# Correction to: A cross-sectional study of community perceptions of stigmatization amongst women affected by UN-peacekeeper perpetrated sexual exploitation and abuse

**DOI:** 10.1186/s12889-022-12507-3

**Published:** 2022-01-24

**Authors:** Samantha Gray, Susan A. Bartels, Sabine Lee, Heather Stuart

**Affiliations:** 1 Department of Public Health Sciences, Queen’s University, Kingston, ON Canada; 2 Department of Emergency Medicine, Queen’s University, Kingston, Canada; 3 Department of History, University of Birmingham, Birmingham, England

**Correction to: BMC Public Health 21, 2295 (2021)**

**https://doi.org/10.1186/s12889-021-12221-6**

Following publication of the original article [1], the authors identified that the given names and family names of all authors were swapped.

The author group has been updated above and the original article has been corrected.
